# Endoscopic Band Ligation for Weight Loss: A Clinical Trial

**DOI:** 10.1007/s11695-024-07609-3

**Published:** 2024-12-20

**Authors:** Mohamed Abeid, Nahla Zaitoun

**Affiliations:** 1 Cairo University, Giza, Egypt; 2 Zagazig University, Zagazig, Egypt

## Abstract

**Background and Study Aim:**

We previously reported the utility of endoscopic band ligation (EBL) in weight loss in a female patient with obesity. This study aimed to evaluate the safety of weight loss using EBL in a larger cohort.

**Patients and Methods:**

This prospective cohort study included 13 female patients aged ≥ 18 years with a body mass index of ≥ 30 kg/m^2^ who were unwilling to undergo bariatric surgery. Patients with obesity-related comorbidities such as diabetes, hypertension, dyslipidemia, and cardiovascular disease were included. Patients with a history of bariatric surgery and those with clinical manifestations of gastroparesis, portal hypertension, liver cirrhosis, and coagulopathies were excluded. Primary study outcomes were technical feasibility and safety, and secondary study outcomes were the percentage of total weight loss, excess weight loss, and the change in gastroparesis clinical symptom index at 1 month after surgery.

**Results:**

No serious complications occurred during the endoscopy or immediately after that. All patients experienced epigastric pain, nausea, and vomiting for the first 3 days, with one patient developing mild hematemesis on the second day that stopped spontaneously and revealed no abnormalities on endoscopy. At 1 month after EBL, the mean percent excess weight loss and total weight loss were 22.3% ± 9.9% and 7.8% ± 2.5%, respectively.

**Conclusions:**

EBL is an effective and safe intervention for obesity management. Further studies with larger cohorts are warranted to comprehensively evaluate of the long-term efficacy and safety of EBL for obesity management.

## Introduction

A major global public health concern is the increasing prevalence of obesity and associated comorbidities, including metabolic dysfunction, steatotic liver disease, and type 2 diabetic mellitus (T2DM), which are leading causes of death worldwide. In the setting of an expected increase in the global obesity rate to over 1 billion adults by 2030 [1–3], bariatric surgery is currently the most effective approach for the management of patients with morbid obesity. However, recent studies have reported unsatisfactory weight loss results following bariatric surgery, including weight loss plateau and, in some cases, weight gain [4]. In addition to several disadvantages, such as anastomotic stenosis and ulceration, gastro–gastric fistula, intestinal obstruction, and surgical leaks, long-term weight loss outcomes of bariatric surgery remain a concern [5]. Therefore, less invasive approaches for weight control, such as endoscopic bariatric therapy, are considered for individuals who are unfit for surgery. In their recent recommendations, the American Society for Gastrointestinal Endoscopy and the European Society of Gastrointestinal Endoscopy suggest the use of endoscopic bariatric therapy with lifestyle modifications for patients with a body mass index (BMI) of > 30 kg/m^2^ and for those with a BMI of 27–29.9 kg/m^2^ with at least one obesity-related medical comorbidity [6].

Traditionally, bariatric endoscopy has been used only to treat the stomach; however, new emerging techniques have allowed for the treatment of the small bowel. Therapies using gastric devices focus on weight loss, with a secondary focus on metabolic disorders, whereas those using small-bowel devices focused on metabolic disorders with or without weight loss. Both therapeutic approaches can be used to prevent or facilitate invasive surgery in patients with obesity [7].

Endoscopic band ligation (EBL), the preferred endoscopic technique for the management of esophageal varices, is a safe approach with low rate of procedural risk [8], illustrating its suitability for obesity management. In the first case report of EBL for weight loss in a female patient with obesity, we previously reported that the patient achieved 7% total weight loss within the first month without evidence of severe complications [9]. In this study, we aimed to assess the utility of EBL for the management of obesity in a larger cohort of patients.

## Materials and Methods

### Patients

This was a prospective cohort study including 13 female patients aged ≥ 18 years with a BMI of ≥ 30 kg/m^2^ at any point during their registration who were unwilling to undergo bariatric surgery due to fear of surgery regarding risks and complications and financial considerations regarding cost and insurance coverage. Patients with obesity-related comorbidities such as diabetes, hypertension, dyslipidemia, and cardiovascular disease were included. Patients with a history of bariatric surgery and those with clinical manifestations of gastroparesis, portal hypertension, liver cirrhosis, and coagulopathies were excluded. After the explanation of the benefits and risks, written informed consent was obtained from all participating patients.

The primary study outcomes were technical feasibility and safety, and the secondary study outcomes were percent total weight loss, excess weight loss, and the change in the gastroparesis clinical symptom index (GCSI) 1 month after surgery. The GCSI score is a composite score of nine symptoms grouped into three categories, namely nausea/vomiting, postprandial fullness, and bloating, and categorized into severity scales (none, very mild, mild, moderate, severe, and very severe). The GCSI score was calculated by averaging the scores of the three symptom categories.

### EBL

**Fig. 1:**
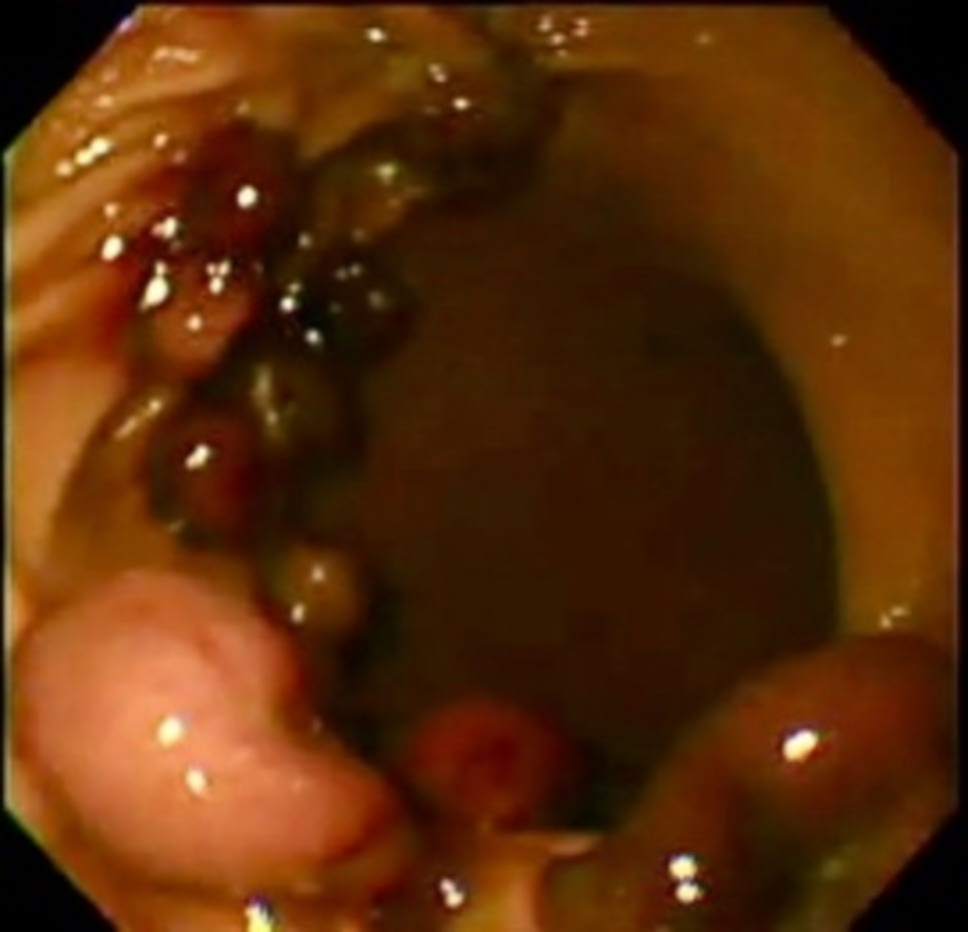
Endoscopy view showing the band ligation in the gastric body

**Fig. 2:**
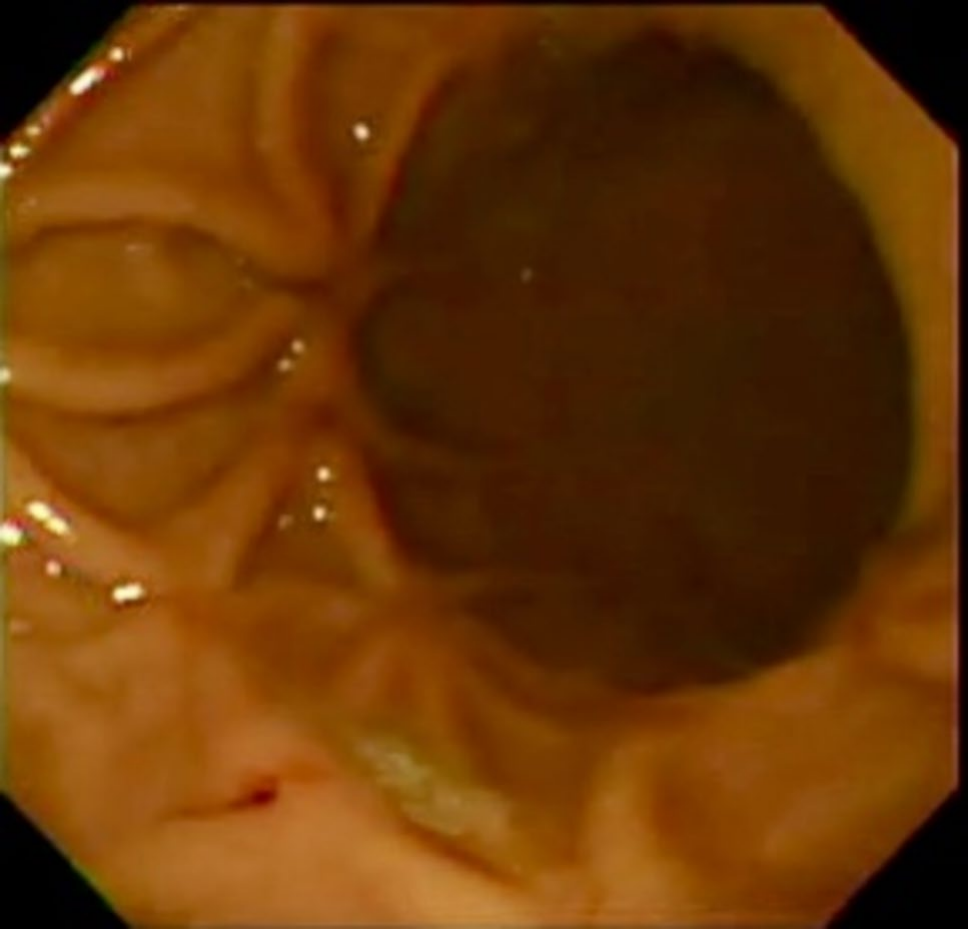
Endoscopic view 1 month after the procedure showing nice linear scars of healed post-band ulcers in the body

**Fig. 3:**
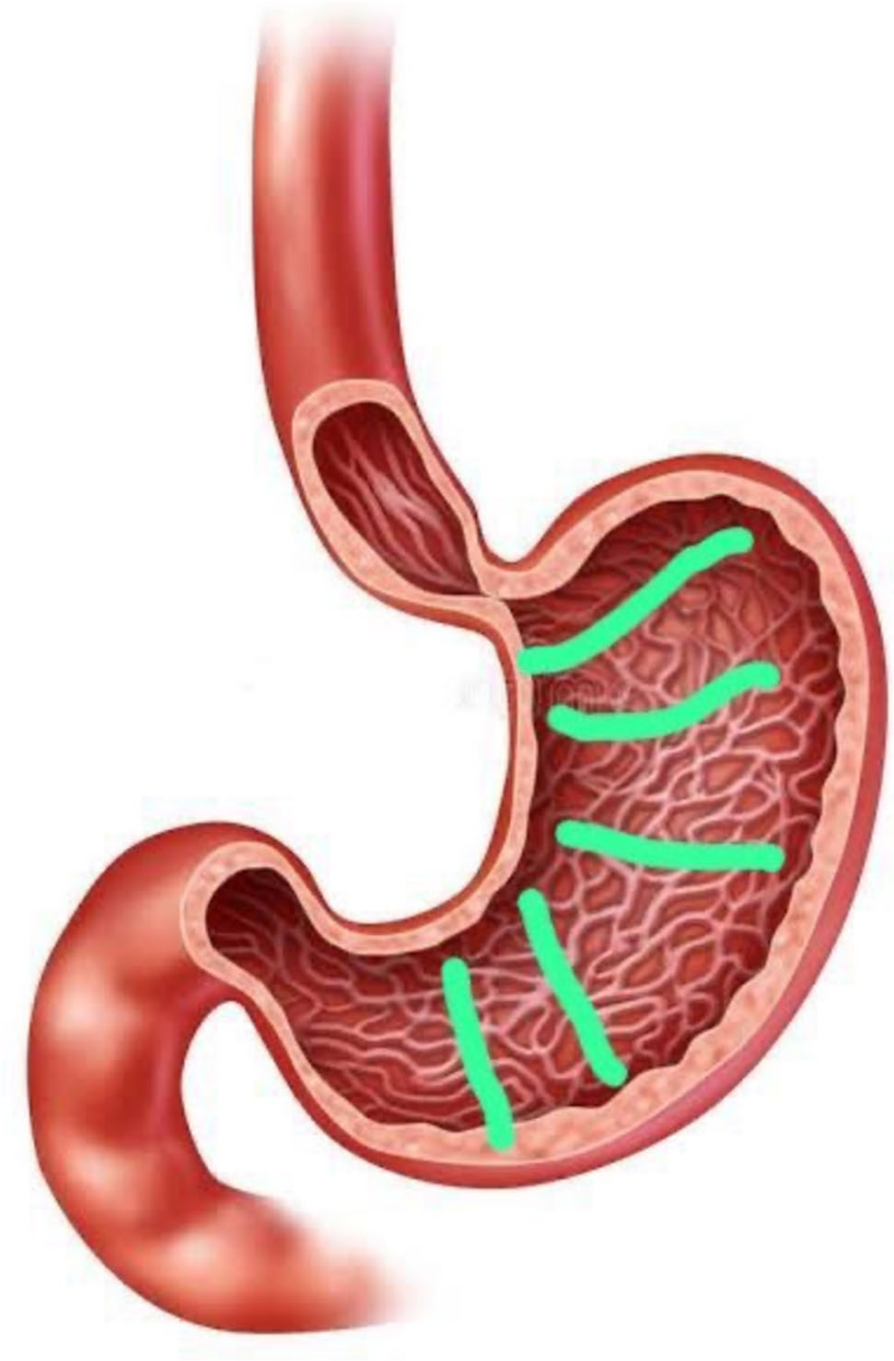
Diagram showing band ligation pattern at gastric body

In all patients, propofol (deep sedation) was used for sedation during endoscopy, and oxygen was used for endoscopic air insufflation. Starting in distal gastric body, five parallel rows of bands were created, for 20–30 bands, and the final row was positioned in the proximal gastric body, where the ligatures were applied. The whole procedure was completed 20–30 min (X` = 24.538 ± 10.102) (Figs. 1, 2, and 3).

During the procedure, all patients were monitored, and no immediate complications were reported. Medications including 40 mg pantoprazole, which was administered twice daily for the first month after the procedure, and antiemetics and antispasmodics were administered on demand to control all issues. The GCSI was determined using postprandial fullness/early satiety, nausea/vomiting, and bloating [10].

Patients were monitored for adverse events before diet resumption. The patients were given a nutritional program, which included the consumption of a liquid diet for 2 weeks, followed by a soft diet for another 2 weeks. Potential complications in all patients were evaluated through endoscopy, which revealed the formation of scars at sites of healed post-band ulcers in the gastric body, causing marginal narrowing of the lumen. Body weight and medical comorbidities associated with obesity were also monitored. The following formulas were used in this study.

### Statistical Analysis

The collected data were entered into a computer and statistically analyzed using the Statistical Package for Social Sciences version 14.0. Qualitative data were presented as frequencies and relative percentages, whereas quantitative data were presented as mean and standard deviation. Differences between any quantitative variables were determined using the *t*-test.

## Results

Following the procedure, all patients recovered well and were discharged within 2–3 h. All patients reported experiencing nausea, vomiting, and epigastric pain during the first 3 days, with one patient experiencing an episode of mild hematemesis on the second day, which stopped spontaneously. However, endoscopy revealed no abnormalities.

As shown in Table 1, in the study cohort of 13 female patients who underwent EBL for weight management, the initial mean body weight was 104.7 ± 17.3 (range, 80–145) kg, the mean BMI was 40.4 ± 4.9 kg/m^2^, and the mean excess weight was 40.0 ± 14.3 kg. Additionally, 61.5% of the patients were in BMI class II, whereas the remaining 38.4% of the patients were in BMI class III.

**Table 1:** Baseline characteristics of the study participants

**Number of patients**	13
**Age, years**	
**Mean ± SD (range)**	37.8 ± 11.1 [17–52]
**Sex**	
**Male (%)**	0
**Female (%)**	13 (100%)
BMI	40.4 ± 4.9
BMI class	
**Class I (25–34.9 kg/m**^**2**^**)**	0
**Class II (35–39.9 kg/m**^**2**^**)**	8 (61.5%)
**Class III (≥ 40 kg/m**^**2**^**)**	5 (38.4%)
**Ideal body weight (kg)**	64.6 ± 4.2
**Excess weight (kg)**	40.0 ± 14.3

*BMI*, body mass index

One month after EBL, the mean body weight (± SD) was 96.6 ± 16.8 kg, corresponding to a mean percent excess weight loss of 22.3% ± 9.9% and a mean percent total weight loss of 7.8% ± 2.5% (Table 2).

**Table 2:** Weight changes and GCSI subscale scores in study participants

Variable	Preoperative	Postoperative	*P* value
Weight, kg, mean ± SD (range)	104.7 ± 17.3 (80–145)	96.6 ± 16.8 (73–133)	0.235
Mean percent excess weight loss	22.3% ± 9.9%		
Mean percent total weight loss	7.8% ± 2.5%		
GSCI score	0		1.5
AEs:			
Nausea and vomiting	*N* = 13% = 100		
Hematemesis	*N* = 1. % = 7.69		

*SD*, standard deviation

No intraoperative or postoperative complications were observed. The GCSI increased from 0 at baseline to 1.5, 1 month after EBL (Table 2).

## Discussion

During EBL, the application of ligatures along the gastric body has been touted as a new well-tolerated and technically feasible approach for inducing weight loss in patients with obesity. The procedure started at the distal gastric body where five parallel rows of bands were created, totaling 20–30 bands, with the final row being positioned at the proximal gastric body containing the ligatures, which induced the formation of linear scars within the healed ulcer in the gastric body, thereby causing marginal narrowing of the lumen. EBL triggered subsequent weight loss similar to that observed with other endoscopic bariatric methods. Specifically, none of the patients had manifestations of gastroparesis, and there were no significant increases in the GCSI subscale scores for nausea/vomiting, bloating, and distention, whereas the GCSI subscale score for fullness and early satiety showed a subsequent increase among the participants.

In the present study, the mean percent excess weight loss and total weight loss 1 month after EBL were 22.3% ± 9.9% and 7.8% ± 2.5%, respectively. In one study comparing intragastric balloon insertion, which limits food breakdown by concentrating on the second phase of digestion, with endoscopic sleeve gastroplasty, intragastric balloon insertion led to a total weight loss of 2.6% in 47 patients, whereas endoscopic sleeve gastroplasty led to a total weight loss of 2.4% in 58 patients 1 month after the procedure [11]. However, despite the achievement of weight loss with intragastric balloon insertion, 17% of the patients reported serious adverse effects. In another study, severe adverse effects resulting in early balloon removal were reported by 28.2% and 10.5% of the patients who underwent intragastric balloon insertion and endoscopic sleeve gastroplasty, respectively [12]; the adverse effects of endoscopic sleeve gastroplasty were more severe and required medical management.

Laparoscopic sleeve gastrectomy induces weight loss through the reduction of stomach capacity with a focus on enhancing satiety and early fullness. Fayed et al. [13] observed a mean weight loss of 8.0% after 1 month. However, the development of postoperative gastroesophageal reflux is a major disadvantage, and endoscopic sleeve gastroplasty is a safer alternative to laparoscopic sleeve gastrectomy. EBL does not appear to induce GERD, although longer and more specific studies are required to confirm this. Additionally, Hadi et al. [14] reported a total weight loss of 5% after 1 month in a cohort of 90 patients who underwent endoscopic sleeve gastroplasty or laparoscopic sleeve gastrectomy, with no significant independent association between the procedure type and total weight loss.

Compared with bariatric surgical approaches, our approach promoted improved weight loss at the end of the first month [15–17]. This finding could have been attributed to the less invasiveness, shorter operative time, fewer adverse events, and technical feasibility of EBL.

In the present study, the patients experienced milder complications compared to those reported for other obesity treatment modalities. Importantly, we did not observe immediate complications during EBL, illustrating its safety. In the present study, the patients complained of nausea, vomiting, and epigastric pain, which were controlled with medical treatment and resolved over 2 days, in contrast to the longer duration of 3–7 days reported by patients who intragastric balloon replacement [18].

Other immediate adverse events, such as upper gastrointestinal bleeding, extragastric hemorrhage, pulmonary embolism, and pneumoperitoneum, were previously reported in patients undergoing laparoscopic sleeve gastrectomy and ESG. However, recent ESG meta-analyses have shown that these AEs are very scarce (< 2%) [19, 20]. Patients with intragastric balloons reported complications such as balloon intolerance requiring early removal, balloon rupture, gastric outlet obstruction, and gastric perforation [16]. EBL did not appear to lead to these complications.

### Limitations of the Study

This study was conducted on a small number of patients without a control group and for a short duration of 1 month for assessing safety and efficacy parameters.

## Conclusion

Our novel EBL approach is an effective and safe short-term intervention for managing obesity.

## Data Availability

No datasets were generated or analyzed during the current study.

## Abbreviations

EBL: Endoscopic band ligation
T2DM: Type 2 diabetic mellitus
BMI: Body mass index
GCSI: Gastroparesis clinical symptom index
SD: Standard deviation
